# Tissierella Praeacuta Infection in the Setting of Chronic Sacral Wounds

**DOI:** 10.7759/cureus.23745

**Published:** 2022-04-01

**Authors:** Johnathone Yang, Danielle Gilbert, Lydia Meece, Aneesa Afroze

**Affiliations:** 1 Internal Medicine, Mercy Medical Center, Des Moines, USA; 2 Infectious Disease, Mercy Medical Center, Des Moines, USA

**Keywords:** sacral ulcer, clostridium hastiforme, gram negative bacteremia, osteomyelitis, maldi-tof-ms, tissierella praeacuta

## Abstract

*Tissierella praeacuta* is a gram negative anaerobe with few documented cases of human infections. This case illustrates a patient who was admitted for infected chronic sacral and ischial decubitus wounds. BACT/ALERT blood culture system using Matrix Assisted Laser Desorption/Ionization-Time of Flight Mass Spectrometry (MALDI-TOF MS) (Bruker Daltonics, Billerica, USA) was employed ultimately identifying *Tissierella praeacuta* as the causative organism. The suspected source of the pathogen was presumed to be from his infected decubitus wounds.

## Introduction

*Tissierella praeacuta* is a gram negative anaerobe that was first discovered in 1908 by Tissier. It is commonly found in the soil and gastrointestinal tract [1]. While initially thought to be its own distinct organism, it was later discovered to be either the same species or very closely related species to *Clostridium hastiforme*, a gram positive rod, as demonstrated by a 99.9% overlap of RNA gene sequence [2]. It is an extremely rare cause of human infections with only a few case reports being found in literature. Of the cases found in medical literature, they have involved chronic sacral wounds, pseudoarthrosis of the femur, pyonephrosis and hepatic abscess, brain abscess, pyometria, osteomyelitis, and gas gangrene of the eyelid [1,3-6]. Treatment consists of beta lactams, rifampin, or metronidazole.

## Case presentation

**Figure 1:**
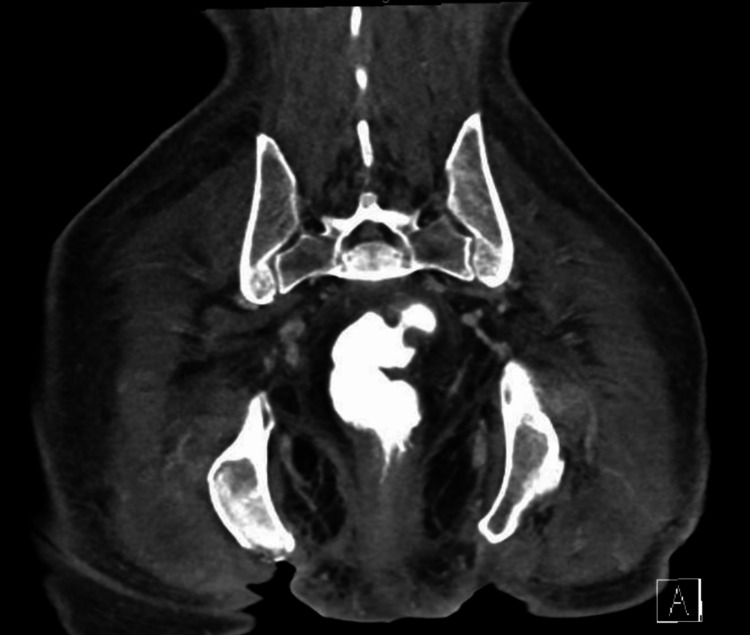
Coronal CT pelvis with contrast demonstrating osteomyelitis of the right ischial tuberosity

A 45-year-old caucasian male with a history significant for paraplegia secondary to spinal cord injury from prior traumatic fall, bilateral above the knee amputation secondary to osteomyelitis, and chronic sacral and bilateral ischial decubitus ulcers was initially admitted for generalized weakness. Family history was unremarkable and social history consisted of tobacco use of one pack per day for over 20 years, one-two alcoholic beverages every couple of months during social gatherings, and no job secondary to his paraplegia. On evaluation in the emergency room, he was afebrile but tachycardic at a heart rate of 110 bpm. His physical exam was remarkable for malodorous purulent discharge coming from his ischial and sacral ulcers with overlying necrotic tissue. He was found to be septic secondary to his infected ulcers with initial labs significant for leukocytosis of 13.4 X 10^3^/uL (reference 4.5-11 X10^3^/uL), C-reactive protein (CRP) of 13.9 mg/dL (reference 0-0.9 mg/dL) and erythrocyte sedimentation rate (ESR) of 110 mm/hr (reference 0-15 mm/hr). Lactic acid was normal at 1.2 mMol/L (reference 0.5-2.2 mMol/L). On further questioning, it was discovered that while he had home wound-care services, he had refused their care for the past month. Blood cultures were drawn and he was initiated on broad spectrum antibiotics with vancomycin 15 mg/kg/dose IV and piperacillin-tazobactam 4.5 g every six hours IV. Given the elevated inflammatory markers and nature of the ulcers, a CT pelvis with contrast was performed to evaluate for osteomyelitis, which demonstrated right ischial osteomyelitis (Figure 1). A transthoracic echocardiogram was also obtained which was negative for any valvular vegetations. General surgery was consulted and performed four rounds of debridement to the bilateral ischial and sacral decubitus wounds down to the bone with a diverting end colostomy placed. Intraoperative cultures from the first three debridements demonstrated pan-sensitive *Pseudomonas aeruginosa,* so he was transitioned from vancomycin and piperacillin-tazobactam to monotherapy with piperacillin-tazobactam. The last round of debridement was performed four weeks into the hospital stay, which resulted in intraoperative cultures growing piperacillin-tazobactam resistant *Pseudomonas aeruginosa *and *Candida albicans/dubliniensis*. Blood cultures were negative, so he was transitioned from piperacillin-tazobactam to levofloxacin 750 mg daily oral for an additional 14 days for a total of six weeks and fluconazole 200 mg daily oral for 10 days. He was then discharged with home wound-care services and follow-up with general surgery and infectious disease.

Two months later, he was re-admitted for complaints of suicidal ideation and worsening bilateral ischial and sacral ulcers. In the emergency room, he was afebrile but tachycardic with a heart rate of 125 bpm. His physical exam again demonstrated malodorous purulent discharge coming from his sacral and bilateral ischial wounds with surrounding erythema and necrotic tissue. He was found to be septic with labs significant for leukocytosis of 21.9 X 10^3^/uL (reference 4.5-11 X 10^3^/uL), lactic acidosis of 4.4 mMol/L (reference 0.5-2.2 mMol/L), procalcitonin of 1.25 ng/mL (reference 0-0.5 ng/mL), ESR of 101 mm/hr (reference 0-15 mm/hr), and CRP of 23.7 mg/dL (reference 0-0.9 mg/dL). Blood cultures were drawn and he was initiated on broad spectrum antibiotics with vancomycin 15 mg/kg/dose IV, levofloxacin 750 mg daily IV, and metronidazole 500 mg every eight hours oral. Of note, he was not started on piperacillin-tazobactam, given the history of piperacillin-tazobactam resistant *Pseudomonas aeruginosa* grown in the prior hospital admission. Given the elevated inflammatory markers and history of ischial osteomyelitis, a CT pelvis with contrast was performed, which demonstrated numerous decubitus ulcers overlying the ischial bone, coccyx, and left greater trochanter without any signs of abscess or osteomyelitis. General surgery was consulted and performed three rounds of debridement of the right superior gluteal wound down to the subcutaneous tissue and mid-ischial and perineal wounds down to the bone. Intraoperative cultures grew *Proteus mirabilis*,* Prevotella bergensis*, and *Bacteroides fragilis*. Blood cultures grew gram negative rods in two out of two bottles and gram positive cocci in one out of two bottles. However, they were unable to be identified until seven days into admission. Ultimately, BACT/ALERT blood culture system using Matrix Assisted Laser Desorption/Ionization-Time of Flight Mass Spectrometry (MALDI-TOF MS) (Bruker Daltonics, Billerica, USA) (resulted in the growth of *Tissierella praeacuta* and *Parvimonas* species. A CT abdomen and pelvis with contrast was then performed to rule out intra-abdominal etiology of *Tissierella praeacuta*, which showed generalized edema without a clear source of infection. Given that the *Parvimonas* species was only in one out of two bottles, this was likely a contaminant so the decision was made by infectious disease on Day 7 of his hospital stay to transition from vancomycin, levofloxacin, and metronidazole to piperacillin-tazobactam 3.375 g every six hours IV for a total of 14 days to treat the *Tissierella praeacuta* bacteremia. Given that he was not a candidate for a wound vac, his wounds were managed with wet-to-dry dressing along with wound-care services. With the IV antibiotic along with wound-care services, his wounds improved with no further discharge along with the resolution of his lactic acidosis, CRP, ESR, and leukocytosis. After 17 days in the hospital, he was safely discharged to a skilled nursing facility.

## Discussion

There are a total of five species belonging to the *Tissierella *genus: *T. praeacuta, T. carlieri, T. creatinine, T. creatinophila, and T. pigra*. While originally thought to have no association with human infections, case reports are slowly surfacing demonstrating the clinical relevance of *Tissierella praeacuta* infections [2]. Despite the emergence of infections from this organism, there is still a lot to learn about this gram negative anaerobe. Although wound cultures were negative, the suspected source of the *Tissierella praeacuta* bacteremia was from his unkempt chronic sacral and ischial ulcers. This was reinforced with a CT abdomen/pelvis with contrast demonstrating no abdominal etiology. This case highlights the difficulty with identification and speciation of this organism. BACT/ALERT blood culture system using MALDI-TOF MS had to be employed for the correct identification of *Tissierella praeacuta*, which was consistent with prior case reports [1,3-6]. This ultimately raises the question of whether or not this organism is truly a rare cause of human infections or if it is actually a common cause of human infections that simply requires additional tools for its identification, particularly given its similarity to *Clostridium hastiforme* [7]. Prior case reports demonstrated sensitivity to beta-lactams, rifampin, or metronidazole, so this patient was treated with piperacillin-tazobactam [8].

## Conclusions

Current literature postulates that *Tissierella praeacuta* is a rare cause of human infections. Given that BACT/ALERT blood culture system using MALDI-TOF MS had to be employed to identify this organism in this case along with other case reports, this raises the question of whether or not this organism is actually a more common cause of human infections that requires additional analytical tools. To shed light on this question, providers will have to consider this gram negative anaerobe in their list of possible pathogens when being presented with a patient with sepsis in the setting of chronic sacral wounds.
